# Effect of Growth Hormone Therapy on a 4-Year-Old Girl with Pfeiffer Syndrome and Short Stature: A Case Report

**DOI:** 10.3390/children9040547

**Published:** 2022-04-12

**Authors:** Min Jeong Jang, Moon Bae Ahn

**Affiliations:** Department of Pediatrics, College of Medicine, The Catholic University of Korea, 222 Banpo-daero, Seocho-gu, Seoul 06591, Korea; 22101241@cmcnu.or.kr

**Keywords:** Pfeiffer syndrome, growth hormone therapy, short stature, fibroblast growth factor

## Abstract

Fibroblast growth factor receptors (*FGFR*s) are expressed in epiphyseal cartilage cells of developing bones and regulate endochondral bone formation with interdependent signaling pathways. Gene mutation in *FGFR*s disrupts the formation of endochondral bony structure by reducing the number of proliferating chondrocytes. Among the syndromes caused by mutation in the *FGFR* gene, Pfeiffer syndrome is a rare inherited disease characterized by acrocephalosyndactyly related to hypertelorism, broad pollex, and hallux. We describe the case of a 4-year-old girl with short stature, advanced bone age, wide thumbs and great toes. The patient was diagnosed with partial growth hormone deficiency and an identified mutation in the *FGFR2* gene. Standard deviation score of her height increased after starting growth hormone therapy. This report will raise awareness of the growth hormone provocation test regardless of bone age in patients with short stature founded *FGFR* gene mutation.

## 1. Introduction

Craniosynostosis is a rare congenital condition involving premature fusion of one or more cranial sutures [1]. Whole exome and genome sequencing has been developed to identify hundreds of mutations implicated in craniosynostosis [2]. The most common mutations in inherited craniosynostosis are fibroblast growth factor receptor (*FGFR*) variants. *FGFR* signaling is present in all organs and tissues in the human body and is involved in homeostasis. In particular, it is known to play an important role in differentiating osteoblasts during bone metabolism. Mutations in the *FGFR* family such as *FGFR1, 2,* and *3* results in unregulated signaling and accelerate the closure of skull sutures. Heterozygous activating mutations of *FGFR2* have been reported as the etiology of several craniosynostosis syndromes. A craniosynostotic disorder associated with *FGFR1* or *FGFR2* gene mutations is known as Pfeiffer syndrome (OMIM #101600) [3].

In 1964, Rudolf Pfeiffer described a syndrome that consisted of acrocephalosyndactyly associated with hypertelorism, normal intelligence, wide thumbs and great toes [4]. Eight cases were reported in three generations of a family, suggesting an autosomal dominant inheritance [5]. Pfeiffer syndrome is the second most common of the acrocephalosyndactyly syndromes and affects one in 100,000 people [6]. The diagnosis of Pfeiffer syndrome is usually determined by the patients’ clinical features. It is divided into three subtypes depending on the expression levels of specific phenotypes. In 1993, Cohen classified and described the three clinical subtypes of Pfeiffer syndrome that are important with regard to prognosis [7]. Patients with type 1, called the “classic” phenotype, have broad thumbs and great toes, variable degrees of brachydactyly or syndactyly, and normal cognitive function [8]. Type 2 reveals severe central nervous system involvement, the prominent cloverleaf skull shape, and intellectual disability. Additionally, patients with type 2 have severe ocular proptosis, elbow ankyloses or synostosis, and wide thumbs and great toes. Patients with type 3 Pfeiffer syndrome have a similar phenotype to that of type 2, except for the cloverleaf skull. However, short stature is not a typical phenotype of any Pfeiffer syndrome subtype.

Short stature is classified as disproportionate and proportionate [9]. The former is rare and is manifested as short limbs or trunks. The latter is the more prevalent type with normal proportionate growth of limbs and trunk. Growth hormone deficiency (GHD) presents as a disorder with proportionate short stature. The growth hormone provocation test is important for patients with short stature, as it identifies the GHD patients who will require extended growth hormone treatment, even after completing linear growth. The fundamental goal of growth hormone treatment is to improve the quality of life and health outcomes for GHD patients from childhood to adulthood. The treatment stimulates the cartilage cells in epiphyseal growth plates to produce insulin-like growth factors (IGFs), which in turn stimulate cartilage cell proliferation and ultimately promote linear growth [10]. Linear growth promotion is expected in children diagnosed with GHD. Other effects of treatment include increased bone density, decreased serum lipids, increased muscularity, and decreased adiposity [11].

To our knowledge, there is no previously reported case in which a patient with Pfeiffer syndrome with short stature was diagnosed with GHD. We report the management, from diagnosis to treatment, in a pediatric patient with Pfeiffer syndrome with GHD.

## 2. Case Report

**Figure 1:**
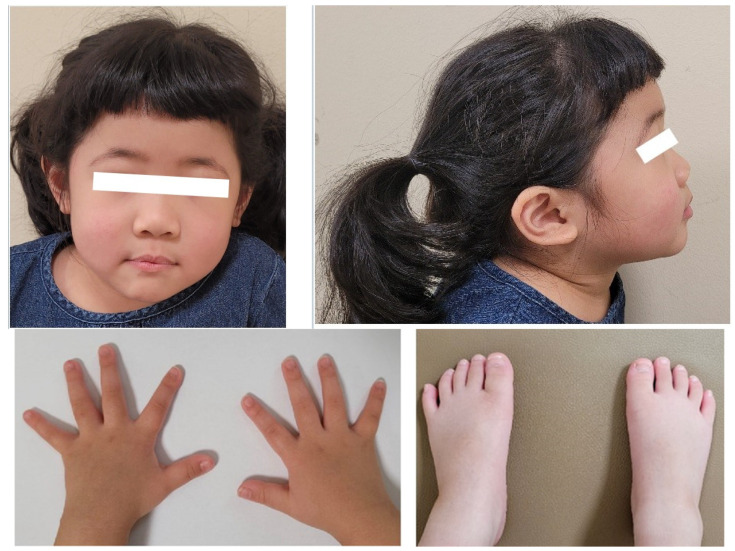
The patient’s face, wide thumbs and great toes.

**Figure 2:**
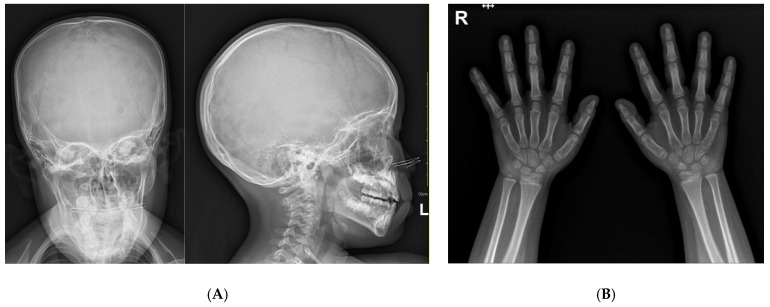
Plain radiography (**A**) Anteroposterior and lateral of skull series. (**B**) Both hand X-rays at chronological age of 4 years and 7 months.

A 4-year-and-7-month-old girl presented with severe short stature below the 3rd percentile. She was born at 40 weeks of gestation with a birth weight of 2.9 kg (standard deviation score (SDS) = −0.75). Her perinatal and neonatal histories were unremarkable. Her parents are non-consanguineous, and her father had no underlying disease. Her mother had previously been under medical therapy for micro-pituitary adenoma. The patient had a healthy 9-month-old brother. No family members had any have current medical problems. On physical examination, her height was 91.1 cm (SDS −3.92), and weight was 15.5 kg (SDS −1.15). Her body mass index was 18.7 kg/m^2^ (SDS 2.05) as normal nutritional status. There were no conspicuous dysmorphic features on her face (Figure 1). Wide thumbs and great toes were observed on both sides (Figure 1) when compared with the other digits. Brachydactyly and syndactyly were not observed in the upper and lower limbs. We also confirmed closure of suture line, anterior and posterior fontanelle in x-ray of her skull series (Figure 2A). At the time of admission, bone age was advanced and measured approximately 7 years according to the Greulich and Pyle atlas (Figure 2B). There were no abnormal findings including serum electrolytes and insulin-like growth factor-binding protein 3 (IGF-BP3) level other than a decrease in the IGF-1 level (108.24 ng/mL, between the 25th and 50th percentiles) and an increase in the prolactin level (27.26 ng/mL, normal range 3.0–18.8 ng/mL) in the initial laboratory tests. Because of the elevated initial prolactin level and her family history, an MRI was performed before confirmation of the pituitary stimulation test results. An arachnoid cyst with a size of 3.5 × 2.6 cm was found in the left middle cranial fossa with magnetic resonance imaging (MRI) of the sella. There were no abnormal lesions in the sellar or suprasellar regions.

The growth hormone (GH)-induced stimulation test was performed after admission. She underwent the test with a combination of two stimulants to assess GH secretion: levodopa (Myung in Pharm, Seoul, Korea; <15 kg: 150 mg) and arginine (Green Cross Well Being, Bundang, Korea; 0.5 g/kg). The two stimulants were tested at intervals of more than 24 h. Blood samples were collected at the time of administration of the stimuli and at 30, 60, 90, and 120 min after administration to measure the peak serum GH concentration level. The peak GH concentration was 7.41 ng/mL after stimulation with levodopa. The GH level was obtained after 30 min of response to arginine; peak GH was 5.58 ng/mL (normal level <10 ng/mL). She was diagnosed as having partial GHD with serum peak GH concentration between 5 and 10 ng/mL on provocation with a combination of two separate stimulation tests [12].

Genetic testing was also conducted to determine the possible causes of short stature other than GHD. There were no abnormal findings in karyotyping and chromosomal microarray. As a result of targeted panel sequencing of 233 genes related to hereditary short stature, c.2398dupT p.(Ser800PhefsTer22) on the basis of the reference sequence NM_022970.3, was classified as a pathogenic variant according to the guidelines of the American College of Medical Genetics and Genomics [13]. The variant had not been previously reported in the *FGFR2* gene, was observed in a heterozygous form and was revealed as the cause of short stature. It can be defined as de novo because the corresponding mutation was not confirmed by the parental genetic test. Although she had wide thumbs and toes bilaterally, her cognitive development was intact. Pfeiffer syndrome is usually characterized by broad, medially deviated thumbs and/or big toes, sometimes with cutaneous syndactyly [14]. Definition of broad thumb and hallux are visible increase in width of the first digit without an increase in the dorso-ventral dimension [15]. We confirmed her hands and feet on the base of the definition. However, Apert syndrome is mainly characterized by symmetrically bilateral syndactyly of the hands and feet. Crouzon syndrome is usually characterized by crouzonoid face apparently distortion of the skull shape and absence of major abnormalities of the hands and feet [16]. This is the reason why we diagnosed her Pfeiffer syndrome rather than Apert syndrome or Crouzon syndrome which are higher incidence of *FGFR2* variant craniosynostotic disorders.She could therefore be clinically diagnosed with Pfeiffer syndrome type I with GHD.

**Figure 3:**
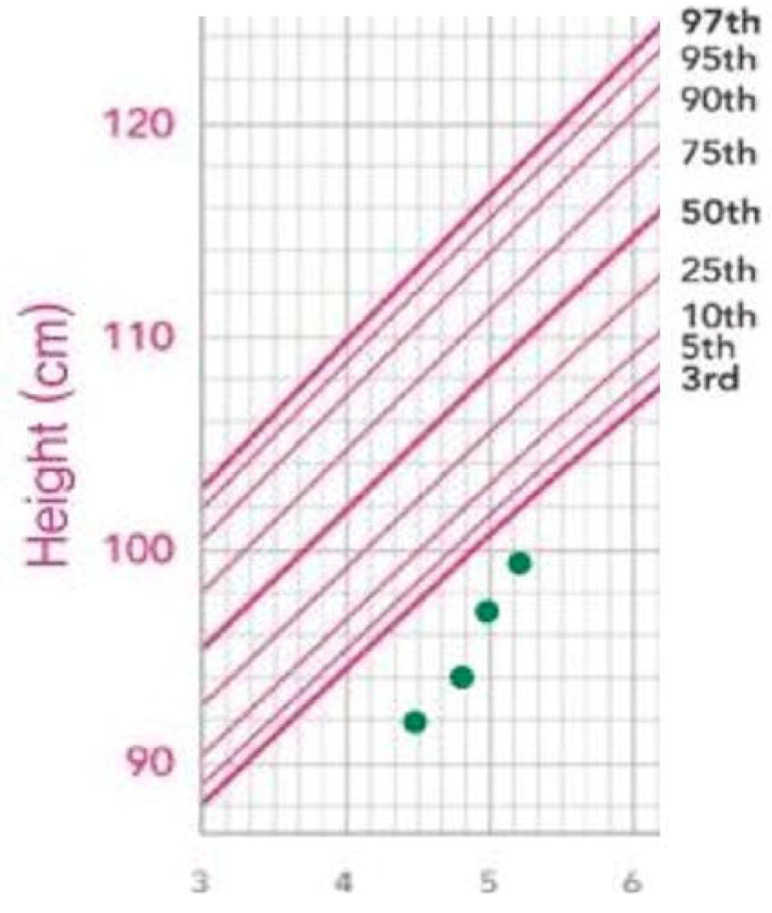
Growth curve of the patient from initial diagnosis to treatment period. The height of the patient was below the 3rd percentile. Modified from Kim, J.H et al. The 2017 Korean National Growth Charts for children and adolescents: development, improvement, and prospects. *Korean J. Pediatr.* 2018, *61*, 135–149, according to the Creative Commons license [17].

Subsequently, recombinant human growth hormone (rhGH) has been administered for the treatment of GHD in units of 0.1 per day according to her weight. The follow-up measured heights were 93.6 cm (SDS −3.66 for age 4 years and 9 months), 98 cm (SDS −2.86 for age 5 years and 2 months), and 99.6 cm (SDS −1.77 for age 5 years, 5 months). Her growth velocity was approximately 8 cm over the eight months since the initiation of GH therapy (Figure 3). No side effects or adverse reactions were observed during the follow-up period.

## 3. Discussion

The patient was admitted with short stature at the first visit, but her bone age was more than 2 years ahead of her chronological age. There were no specific findings other than broad thumbs and great toes. A GH provocation test revealed a diagnosis of GHD. However, a genetic evaluation was conducted to evaluate the possible additional causes of her short stature. Karyotyping and chromosome microarray was normal. A de novo mutation of *FGFR2* gene, which causes craniosynostosis, was observed in the next-generation sequencing test. In addition, there were not observed another 232 gene mutations leading to short stature such as *ACAN, FGFR1, FGFR3, IGF1, IGF1R, PTPN11, SOS1* and *SHOX* included from panel of next-generation sequencing test. A diagnosis of Pfeiffer syndrome with partial GHD was made on the basis of the clinical, genetic, and laboratory results. rhGH treatment was initiated and doctors have been monitoring her growth and development as well as any complications which could arise owing to treatment.

As mentioned, short stature is not a primary clinical feature of craniosynostosis including Pfeiffer syndrome. However, *FGFR2* gene mutation has been reported as a cause of craniosynostosis in patients with short stature. Fibroblast growth factors (FGFs) and their receptors are expressed in epiphyseal cartilage cells of developing bones. FGFs regulate endochondral bone formation with interdependent signaling pathways [18]. Therefore, activation of mutations in *FGFR*s disrupts endochondral bone formation by reducing the number of proliferating chondrocytes [19]. Signaling through *FGFR2* regulates the proliferation of stem cells, affecting a variety of systems including the growth of osteoblasts and chondrocytes [20]. The *FGFR2* mutation in the metaphysis of mice reduces the bone mass due to decreased trabecular bone volume and shortened growth plates in the long bones. The mutation directly affects endochondral ossification, resulting in long bone growth retardation. Molecularly, the p38 and Erk1/2 signaling pathways have been reported to partially mediate the effects of *FGFR2* mutation on long bone development [21]. It was confirmed that the axial length of *FGF*R2 mutant mice was relatively significantly shorter than that of wild mice. Additionally, previous studies about *FGFR2* mutation proved both gain and loss of function [22]. Premature fusion and growth of skull or advanced bone age is revealed due to enhanced proliferation and differentiation. However, decreased proliferation and differentiation affects decreased trabecular bone volume, shortened growth plates in the long bones and lead to be short stature.

The following are examples of syndromes other than Pfeiffer syndrome in which the *FGFR2* mutation has been associated with clinically short stature and craniosynostosis (Table 1): Apert syndrome [23], Crouzon syndrome, and Bent bone dysplasia syndrome [19,20,21]. Among them, two patients with Crouzon Syndrome with GHD were reported in twenty years. An 11 years 5 months old boy diagnosed with Crouzon syndrome with short stature (SDS −5.0) was reported in 2010 [24]. Due to a progressive increase in ventricular size and rising intracranial pressure caused by cerebellar tonsil herniation with hydrocephaly, he received ventricular shunting and frontal advancement at 22 months. He had brachycephaly, maxillary hypoplasia, severe ocular proptosis with hypertelorism, and normal cognitive function. His bone age was 4.5–5 years according to the Greulich and Pyle atlas. Peak GH levels from the GH stimulation test were 4.57 uIU/mL in the insulin test and 2.63 uIU/mL in the clonidine test. Although chromosome analysis was normal in the G-banding karyotype, a point mutation of Cys278Phe in exon IIIa of the *FGFR2* gene was identified in molecular analysis. The patient’s growth velocity was increased to 5 cm per year with rhGH therapy.

Most patients with short statures reveal a delayed bone age as well as endocrine disease, chronic disease, malnutrition, and frequently, idiopathic short stature [25]. Conversely, advanced bone age is most commonly associated with tall stature, presenting with precocious puberty and hyperthyroidism. It is natural to consider the GH provocation test in children with short stature accompanied by delayed bone age in patients like the one with Crouzon syndrome. Other endocrine evaluations such as the GnRH stimulation test or genetic evaluation can be prioritized over the GH provocation test because the combination of short stature and advanced bone age is much less common [26]. However, as in this case of Pfeiffer syndrome with short stature, the GH stimulation test should not be excluded because of the advanced bone age. In other words, patients who require growth hormone treatment should not be overlooked by the clinic.

As an additional example, it is debatable whether GH therapy should be applied in the case of achondroplasia, an *FGFR3* mutation with short stature. Achondroplasia is also not associated with GHD. An 8-year-old girl diagnosed as having achondroplasia with GHD was reported in 2017 [27]. Her height was below the 3rd percentile and her bone age was approximately 6 years and 10 months according to the Greulich and Pyle atlas. She was diagnosed with partial GHD, similar to our case. Her growth velocity after starting rhGH treatment was 5 cm in 6 months. She did not experience any adverse effects under GH therapy, nor exhibited abnormal findings in the fasting glucose and thyroid function test results. Based on our case and others reported thus far, GH replacement is suggested as the treatment of choice based on the previous cases of *FGFR* gene mutation with GHD. 

If the following limitations of our case are corrected, the efficacy of GH therapy may be further reinforced. Longer-term tracking of growth velocity is necessary to obtain information on the effect of growth hormone therapy, as our follow-up period was relatively short. Moreover, precise monitoring is required for long-term adverse effects that may occur due to growth hormone therapy.

We conclude three points: First, short stature, as well as GHD, may occur even in patients with *FGFR* gene mutations in which short stature is not the predominant phenotype. Second, rhGH treatment might be helpful to patients diagnosed with an *FGFR* gene mutation with GHD. To avoid overlooking such patients, clinicians should consider a GH provocation test in patients with a suspected syndromic disease with short stature, regardless of bone age.

## Figures and Tables

**Table 1:** FGFR2 mutation associated craniosynostosis syndromes with short stature (* the patient).

	Pfeiffer Syndrome	Apert Syndrome	Crouzon Syndrome	Bent Bone Dysplasia Syndrome
Prevalence	1:100,000	1:60,000	1:60,000–25,000	<1/1,000,000
Inheritance	Autosomal dominant	Autosomal dominant	Autosomal dominant	Autosomal dominant
Gene	FGFR1, FGFR2	FGFR2	FGFR2, FGFR3	FGFR2
Mutation	Ala314Ser, Asp321Ala, Cys278Phe, Cys342Tyr, Cys342Arg, Cys342Ser, Cys342Trp, Ser351Arg, Trp290Cys, Tyr340Cys, Thr342Pro, Val359Phe Ser800Phe *	Ser252Trp, Ser252Phe, Pro253Arg	Ala344Gly, Cys278Phe, Cys342Tyr, Cys342Arg, Cys342Phe, Cys342Ser, Cys342Trp, Gly338Arg, Ser267Pro, Ser347Cys, Ser354Cys, Trp289Gly, Tyr290Gly, Tyr328Cys, Tyr340His	Met391Arg Tyr381Asp
Extracranial Symptoms	Frontal bossing, Hypertelorism, Wide thumbs and great toes Brachydactyly or Syndactyly	Brachyturricephaly, Broad forehead, Depressed nasal bridge Syndactyly	Flattened forehead, Proptosis, Hypertelorism, Beaked nose	Hepatosplenomegaly, Bell-shaped thorax, Brachydactyly, Bent long bones Osteopenia

## Data Availability

Clinical data could be provided under author’s permission without undue reservation.
